# Early Growth Patterns of *Bacillus cereus* on Potato Substrate in the Presence of Low Densities of Black Soldier Fly Larvae

**DOI:** 10.3390/microorganisms11051284

**Published:** 2023-05-15

**Authors:** Matthew Moyet, Hailey Morrill, Daniella Leal Espinal, Edward Bernard, Andrei Alyokhin

**Affiliations:** 1 School of Biology and Ecology, University of Maine, Orono, ME 04469, USA; matthew.moyet@maine.edu; 2 Department of Molecular and Biomedical Sciences, University of Maine, Orono, ME 04469, USA

**Keywords:** *Bacillus cereus*, *Hermetia illucens*, black soldier fly, bioremediation

## Abstract

*Bacillus cereus* is a common and ubiquitous bacterium that can cause foodborne illnesses in humans and other animals. Common methods of contact between foodborne pathogens and their victims include exposure through contaminated food or food containment products. Using larvae of black soldier flies, *Hermetia illucens*, for biological conversion of wastes into components of animal feeds is a rapidly growing technology. However, contamination of larval biomass with pathogenic microorganisms may challenge its use on an industrial scale. We conducted laboratory experiments to test the effects of the black soldier fly larvae developing on simulated potato waste substrate on *B. cereus* abundance. We observed a general increase in the number of colony-forming units and concentration of *hblD* - gene when the larvae were present in the substrate, although the effect was modulated by larval densities and time since inoculation. It is possible that starch breakdown by black soldier fly larvae may provide a beneficial environment for *B. cereus*. Our results differ from the suppression in the presence by black soldier fly larvae reported for several other bacterial species and highlight the importance of taking proper food safety measures when using this technology.

## 1. Introduction

Increasing global population and resultant economic efforts have led the search for alternative protein sources that provide similar nutritional profiles to current products such as beef or poultry. This diversification of protein sources has led to the introduction of novel food items, such as insect protein, into commercial food settings [1,2]. Using insect protein has also gained considerable attention in mainstream animal feed markets. Recent advances have focused on the incorporation of black soldier fly larvae, *Hermetia illucens*, into various industrial applications due to their high bioconversion rates and abundant protein within biomass. However, there is increasing concern regarding consumer safety from contaminants such as toxic chemicals or pathogenic bacteria that black soldier fly larvae may be exposed to during rearing [3,4]. However, research of food and feed safety issues surrounding widescale use of insect meal is still insufficient to address this issue [5,6].

Previous studies have found that the antimicrobial properties of black soldier fly larvae are capable of suppressing primarily Gram-negative bacteria, although some Gram-positive bacteria such as *Staphylococcus aureus* can also be reduced [7,8,9]. Research focused on how endospore forming bacteria interact with the environment in the presence of larvae is minimal, which raises concerns given how endospore production exacerbates microbial activity. A previous study has indicated endospore forming microbes, such as members of the *Bacillus* and *Clostridium* genera, are reduced by the presence of black soldier fly larvae, yet the corresponding endospore ecology remains unreported [10].

Microbes that lead to foodborne illnesses are a persistent threat to the health and safety of consumers, which also makes them detrimental to economic well-being. Sources for these illnesses often involve food waste products bearing rich microbiomes. Several bacteria responsible for these illnesses include species within *Enterobacteriaceae,* such as *Salmonella* sp. and *Shigella* sp., as well as members of *Bacillaceae,* such as *Bacillus cereus*. Differences in *B. cereus* species physiology allow for two primary infection pathways in hosts: emetic and diarrheal gastrointestinal disease [11]. Host infection with emetic strains results from the production of cereulide, which leads to disruptions in cellular ionic activity and subsequent illness responses [12]. Infections involving diarrheal host responses involve the use of hemolysins that disrupt cell membrane permeability and lead to fluid spillage and accumulation within the gastrointestinal tract [12]. Additional concerns regarding *B. cereus* activity also focus on the endospore-forming capabilities of several *Bacillus* sp., which allow for dormancy periods followed by subsequent infections [13,14].

The versatility of this foodborne bacterium in habituative settings has caused widespread public health and food safety concerns across the globe. Common methods of contact with foodborne pathogens include exposure through contaminated food or food containment products [3]. On certain food substrates, such as potatoes, the possibility of *B. cereus* proliferation can increase due to the amylase activity of *B. cereus* that allows for starch breakdown and enhanced microbial proliferation [15,16]. *Bacillus cereus* is also closely related to bacteria, such as *Bacillus anthracis* and *Bacillus thuringiensis*, both of which depend on similar metabolic strategies as *B. cereus* and can potentially exacerbate their presences in the environment [6]. In addition to the concern for human biosafety, variants of *B. cereus* have also been reported to excrete toxins capable of harming other mammalian and arthropod organisms, although their ability to parasitize black soldier flies is unknown. The overarching theme of the present study is to assess how the presence of black soldier fly larvae in the potato substrate influences survival and growth of the endospore-forming *B. cereus*.

## 2. Materials and Methods

### 2.1. Insect Origins

Black soldier fly larvae were obtained from a commercially available colony (Symton BSF Company, College Station, TX, USA) kept on the Gainesville diet (50% wheat bran, 30% alfalfa, and 20% cornmeal, *v*/*v*) [17]. In our laboratory, the colony was maintained in 8 qt. ventilated plastic containers at 25 °C. Larvae were supplied unsterilized potatoes ad libitum for 48 h to reduce possible impacts of dietary changes during experiments. In the beginning of each trial, the second and third instars were removed from the substrate using featherweight forceps. All treatments were replicated three times.

### 2.2. Bacillus cereus Strain

A laboratory isolate of *Bacillus cereus* was used for the experiments described below. It was confirmed as *Bacillus cereus* through extensive biochemical testing [18], growth and differential characteristics (lecithinase-positive, mannitol-negative) on Oxoid *Bacillus cereus* agar (Oxoid, Hampshire, UK) [18], and molecular characterization of the hemolysin gene (*hblD*) [19]. This particular isolate was negative for the emetic toxin (*ces*), as confirmed by PCR [20]. It was also confirmed to lack the insecticidal toxin regulator (*XRE*) present in isolates of *Bacillus thuringiensis,* a closely related species [21].

### 2.3. Sterilization and Preparation of Materials

One-pint (473 mL) glass Mason jars, metal lid screw bands, and cheesecloths were sterilized by autoclaving (121 °C, 18 psi, 30 min). Organic russet potatoes (Nature’s Promise; Scarborough, ME, USA) were purchased in three-pound bags from the local grocery store for use as substrate. These potatoes were washed with deionized water and sterilized by autoclaving (121 °C, 18 psi, 30 min). Following sterilization, potatoes were cooled for 30–45 min and mashed with a sterile scoopula. Processed potatoes were weighed and put into pre-sterilized 473 mL Mason jars, with 165 g per jar. To confirm sterility, five jars were selected at random and swiped with a sterile cotton swab. The swabs were streaked onto trypticase soy agar plates and incubated for 24 h in an environmental chamber at 37 °C with 95% relative humidity to assess microbial growth.

To reduce their contamination with substrate, the larvae were rinsed with 700 mL of sterile distilled water and placed on top of dry paper towels to dry. Further sterilization was not done to avoid damaging the larvae or creating an environment that would not represent in vivo studies. Clean and dry larvae were weighed and counted. At the beginning of the experiments, an average larva weighed ca. 7.3 × 10^−2^ ± 0.1 g (mean ± SD). After adding the components necessary for each treatment group, each jar was covered in sterile cheesecloth and secured using a sterile metal screw band. Jars were kept in a chemical fume hood with the sash lowered at room temperature and with natural daylight.

### 2.4. Experimental Setup

Two experiments were conducted to investigate the interaction between black soldier fly larvae and *Bacillus cereus*. Each experiment utilized sterilized potato as a substrate and addition of *B. cereus*, black soldier fly, or neither. There were two major differences between the experiments. The first utilized a higher starting *B. cereus* concentration to account for an increased larval density. The second incorporated the same larval density in all treatments containing BSF, a lower starting *B. cereus* concentration, and an additional treatment to explore the native commensalistic burden of *B. cereus.*

In the first experiment, the following four experimental treatment groups were created: potato only (control), potato with *B. cereus* (BC), potato with 50 black soldier fly larvae and *B. cereus* (BC + 50 BSFL), and potato with 100 black soldier fly larvae and *B. cereus* combined (BC + 100 BSFL). *B. cereus* was inoculated into 50 mL of trypticase soy broth and incubated for 18–20 h at 37 °C with 95% relative humidity. The culture was then diluted to an optical density (OD_600_) of 0.35, which corresponded to the starting *B. cereus* concentration of approximately 1.0 × 10^8^ CFU/mL. This dilution was added to appropriate potato substrates at a rate of 0.01 mL/g culture based on the previously established relationship of volume (0.01 mL) to substrate weight (g) [10].

In the second experiment, the following four experimental treatment groups were created: potato only (control), potato with *B. cereus* (BC), potato with 50 black soldier fly larvae (50 BSFL), and potato with *B. cereus* and 50 black soldier fly larvae combined (BC + 50 BSFL). The incorporation of the potato with 50 black soldier fly larvae treatment was based on a desire to ascertain the quantitative contribution of possible gut-associated *Bacillus cereus* to the bulk substrate during larval feeding. Larval density was similarly limited to 50 individuals per jar because results of the first experiment showed diminished total bacterial and *B. cereus* growth for 100 individuals per jar (see Results below). *B. cereus* was inoculated into 50 mL of trypticase soy broth and incubated for 18–20 h at 37 °C with 95% relative humidity. This culture was diluted to an optical density (OD_600_) of 0.30, which corresponded to the starting *B. cereus* concentration of approximately 4.0 × 10^7^ CFU/mL. After seeing an apparent synergistic effect in the first experiment, we chose to reduce the starting inoculum to reduce the possibility of bacterial overgrowth during plating [10]. This dilution was added to appropriate substrates at the volume to weight ratio of 0.01 mL/g as utilized in previous setups [6,10]. BSFL used were sourced from the same colony utilized in the previous experiment and kept under similar rearing conditions. All treatments were produced in triplicate.

### 2.5. Sampling and Plating of Substrate

Substrates were thoroughly mixed within the jars and three samples of 5 g each were taken aseptically from each jar on day 2 and day 5 of the first experiment and on day 2 and day 4 of the second experiment. These time points were selected based on multiple factors and prior preliminary experiments on the growth kinetics of larvae on potato as a substrate. In previous experiments, feeding for 7 days or more on potato resulted in escape behavior of larvae (unpublished data), reduction in the substrate biomass, and many plates with bacterial overgrowth following dilution beyond 10^−9^. The latter phenomena were especially observable when more than 50 larvae were present in a jar. Each 5 g sample was inspected to ensure the absence of larvae, added to 45 mL of sterile water, and gently swirled (50–100 rpm) for 5–10 min using a benchtop orbital shaker. During the first experiment, the batches of three flasks were then serially diluted to obtain concentrations of 10^−2^ through 10^−8^ and plated onto both trypticase soy agar (TSA) and Oxoid *Bacillus cereus* agar (BCA) for enumeration of total bacterial colonies and *Bacillus*-specific colonies, respectively. During the second experiment, trypticase soy agar was foregone to focus on the specific growth kinetics of *B. cereus* in response to the presence or absence of black soldier fly larvae, rather than as a function of the overall bacterial community. In this experiment, the triplicate batches of flasks were diluted to obtain concentrations of 10^−2^ through 10^−7^, and only Oxoid *Bacillus cereus* agar was used. All bacterial plates were incubated for 24 h at 37 °C. Colony counts were recorded as the number of colony-forming units per milliliter (CFU/mL) of substrate. Total bacterial counts were collected from TSA, while only *B. cereus*-consistent colonies (lecithinase-positive, mannitol-negative) were enumerated from BCA. Data from the two lowest dilutions were averaged for each experiment and standardized to 10^7^ for the first experiment and to 10^6^ for the second experiment.

### 2.6. Extraction and Preparation of Genomic Material

All substrate samples were collected and stored at −20 °C prior to being subjected to DNA extraction. Extractions were performed using the DNeasy PowerFood Microbial Kit (Qiagen, Germantown, MD, USA), which is made for total microbial DNA extraction from food sample matrices. Extractions were conducted following the manufacturer’s specifications without prior enrichment of substrate samples. The quality of the extracted genomic DNA (gDNA) was assessed on a 0.8% agarose gel at 35V for a 4-h period. Nucleic acid was purified from the substrate with 50 black soldier fly larvae in the second experiment based on the results obtained during the first.

### 2.7. Diagnostic PCR Setup

Diagnostic (non-quantitative) PCR was performed on extracted substrate DNA for detection of *B. cereus*. Reaction conditions were as follows: 10 min of initial denaturation at 95 °C; followed by 35 cycles of 95 °C for 30 s, 52.2 °C for 30 s, and 72.0 °C for 30 s; and a final elongation of 10 min using Promega GoTaq green master mix (Promega, Madison, WI, USA).

Primer sets (Table 1 and Table 2) were selected based on published information targeting two virulence genes associated with *B. cereus*: β-hemolysin (*hblD*) and cereulide synthase (*ces*) [19,20]. Additionally, a primer set targeting the transcriptional regulator of the insecticidal toxin (*XRE*) found only in *B. thuringiensis* was tested against both *Bacillus cereus* genomic DNA and DNA extracted from substrate samples. This was intended to corroborate the enumeration of bacteria grown on selective media in both experiments as *B. cereus* and not *B. thuringiensis*, since the two are impossible to visually tell apart on Oxoid growth medium [21]. Negative controls for both diagnostic and quantitative PCR consisted of primers combined with PCR reagents and nuclease-free water. Positive controls included gDNA isolated from an undiluted *B. cereus* culture. PCR products were visualized by gel electrophoresis using 2% agarose gels and using a 1 kb DNA ladder (New England Biolabs, Ipswich, MA, USA). The electrophoresis conditions were 50V for approximately 2 h. Gel was stained prior to imaging by submersion in a 1 ng/mL solution of ethidium bromide for 10 min.

### 2.8. Quantitative (qPCR) Setup

Quantitative PCR reactions were conducted using 4 μL of extracted DNA in a 20 μL reaction volume of SsoAdvanced Universal Probes Supermix (Bio-Rad, Hercules, CA, USA), in a CFX96 Touch real-time PCR detection system (Bio-Rad, Hercules, CA, USA). All qPCR reactions followed a sequence of 10 min of initial denaturation at 95 °C, followed by 35 cycles of 95 °C for 30 s and 52.2 °C for 30 s with an elongation step at 72 °C for 30 s. Nuclease-free water was added in place of the template DNA in negative controls, and undiluted *B. cereus* genomic extracts were used in positive controls. A standard curve was constructed of 10-fold serial dilutions from a gDNA extract of *B. cereus* culture that was standardized to an OD_600_ of 0.35, which is approximately 1 × 10^8^ CFU/mL and corresponded to a DNA concentration of 6.3 ng/μL. The concentration of the standardized *B. cereus* culture using a Thermo-Fisher Scientific Nanodrop One Spectrophotometer (Thermo-Fisher Scientific, Waltham, MA, USA). The limit of detection for both diagnostic and quantitative PCR was found to be 1 × 10^6^ CFU/mL, or 0.063 ng/μL, for samples in the presence of the potato substrate. All measurements on treatment biological replicates (jars; described above), standards, and controls were performed in triplicate.

### 2.9. Statistical Analysis

Analysis of all data was conducted using SAS University Edition Software (SAS Institute, 2021, Cary, NC, USA). Data distribution was assessed using the Shapiro–Wilk test (PROC UNIVARIATE) and found to be non-normal (*p* < 0.01). As a result, they were transformed using rank transformations (PROC RANK) [22,23] for further analyses. Untransformed data are presented as box plots in the Results Section for ease of interpretation.

Treatment, day of sampling, and interactive treatment by day of sampling effects were analyzed using repeated measures ANOVAs (PROC MIXED). When overall treatment effects were significant (*p* < 0.05), mean separation was carried out using Tukey’s honestly significant difference (HSD) tests. When treatment by day of sampling interactions were significant, treatment effects on each of two sampling days were tested individually using SLICE option (PROC MIXED).

## 3. Results

### 3.1. Colony Count Results Using Selective and Non-Selective Media

**Figure 1:**
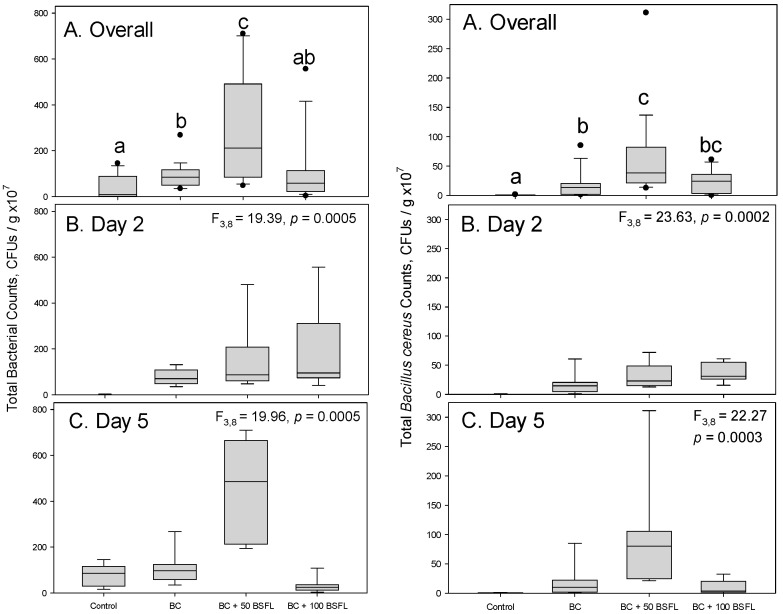
Bacterial counts on TSA plates in the first experiment. Left pane—total bacteria, right pane—*Bacillus cereus.* Control—potato only, BC—potato with *Bacillus cereus*, BC + 50 BSFL—potato with *B. cereus* and 50 black soldier fly larvae combined, BC + 100 BSFL—potato with *B. cereus* and 100 black soldier fly larvae combined. (**A**) Overall counts during the entire experiment; (**B**) counts on day 2 of the experiment; (**C**) counts on day 5 of the experiment. Box plots followed by the same lowercase letters indicate treatments that were not significantly different from each other (Tukey tests, *p* < 0.05). Separate F and *p* values for the two sampling days were obtained using the SLICE option (PROC MIXED, SAS Institute, 2021, Cary, NC, USA).

In the first experiment, total bacterial counts were significantly affected by treatment (df = 3,8, F = 21.40, *p* < 0.0004), day of sampling (df = 1,8, F = 6.43, *p* = 0.0350), and by their interaction (df = 3,7, F = 17.87, *p* = 0.0007). Overall, control jars had the lowest bacterial abundance, while inoculation of potato substrate with both *B. cereus* and 50 black soldier fly larvae resulted in dramatically higher numbers of CFUs compared to other treatments (Figure 1A, left pane). At the same time, inoculation with both *B. cereus* and 100 black soldier fly larvae resulted in bacterial abundance similar to that observed in the uninoculated control (Figure 1A, left pane). On day 2, all treatments inoculated with *B. cereus* had more CFUs compared to the uninoculated control, which had very low bacterial abundance (Figure 1B, left pane). On day 5, there was a dramatic increase in bacterial counts in the presence of 50 black soldier fly larvae, but not in the presence of 100 black soldier fly larvae (Figure 1C, left pane). The number of colonies also increased in the uninoculated potato substrate. Bacterial abundance remained fairly stable in the inoculated substrate without the larvae and declined slightly in the inoculated substrate containing 100 larvae (Figure 1C, left pane).

The treatments also had a significant effect on *B. cereus*-specific colony counts (df = 3,8, F = 36.19, *p* = 0.0001), but there was no statistical significance between the days when each treatment was measured (df = 1,8, F = 0.02, *p* = 0.8806). The interactive effect of treatment and day of sampling, however, was significant (df = 3,8, F = 7.46, *p* = 0.0105). Minimal growth of *B. cereus* colonies occurred in the absence of both *B. cereus* and black soldier fly larvae inoculation, yet proliferation of *B. cereus* within the substrate was observed within two days post inoculation, especially when the larvae were present (Figure 1, right pane). The highest number of CFUs was observed in potato substrate inoculated with both *B. cereus* and 50 black soldier fly larvae. Increasing the number of larvae to 100 resulted in the overall bacterial counts being intermediate between the substrate inoculated with *B. cereus* but no larvae and the substrate inoculated with both *B. cereus* and 50 larvae (Figure 1A, right pane). On day 2 after inoculation, both treatments inoculated with larvae had higher numbers of CFUs (Figure 1B, right pane). However, by day 5, the abundance of *B. cereus* significantly increased in the substrate containing 50 larvae but became comparable to that in the inoculated substrate without larvae for the substrate containing 100 larvae (Figure 1C, right pane).

**Figure 2:**
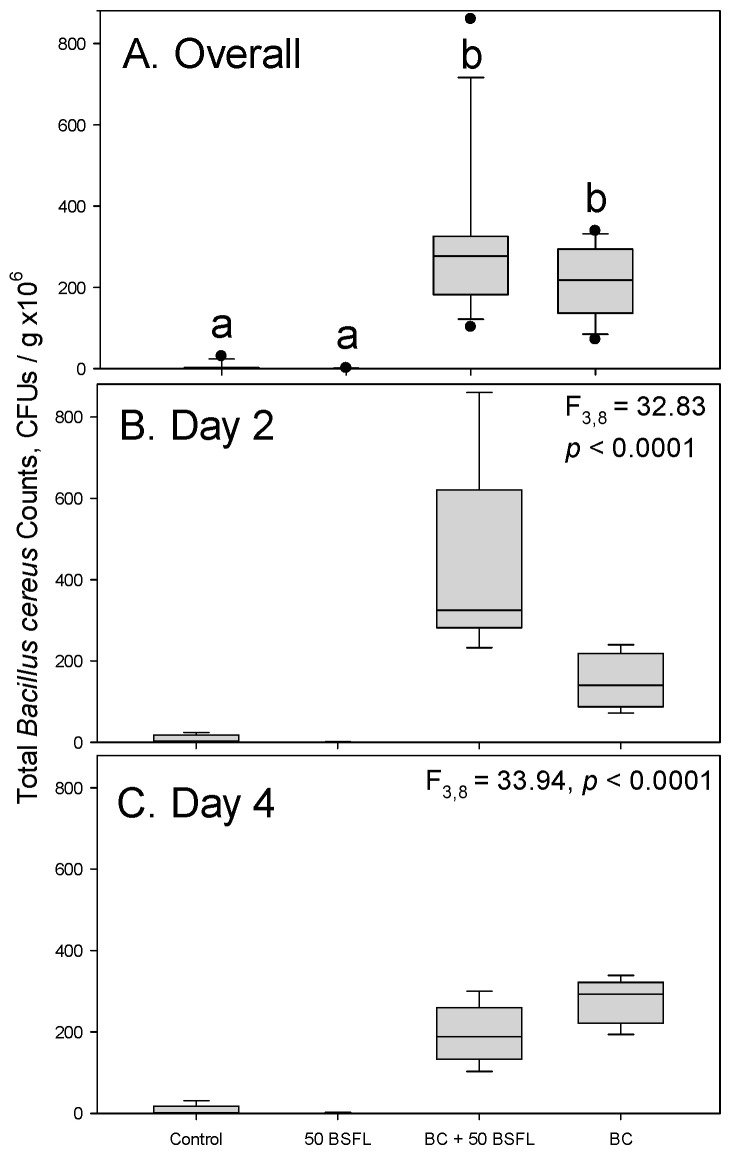
Total *Bacillus cereus* colony counts on Oxoid selective agar in the second experiment. Control—potato only, BC + 50 BSFL—potato with *B. cereus* and 50 black soldier fly larvae combined, BC—potato with *Bacillus cereus*. (**A**) Overall counts during the entire experiment; (**B**) counts on day 2 of the experiment; (**C**) counts on day 4 of the experiment. Box plots followed by the same lowercase letters indicate treatments that were not significantly different from each other (Tukey tests, *p* < 0.05). Separate F and *p* values for the two sampling days were obtained using the SLICE option (PROC MIXED, SAS Institute, 2021, Cary, NC, USA).

In the second experiment, treatments had a significant effect on *B. cereus*-specific colony counts (df = 3,8, F = 42.83, *p* < 0.0001). No statistical significance was detected between the days of the experiment (df = 1,8, F = 0.01, *p* = 0.9301). The interaction between the treatment and the day was significant (df = 3,8, F = 7.29, *p* = 0.0112). Both treatments inoculated with *B. cereus* had considerable numbers of CFUs present, while virtually no bacterial growth was detected in the absence of inoculation with that bacterium (Figure 2A). There was an evident exacerbation of growth of *B. cereus* colonies in the samples collected on day 2 when black soldier fly larvae were present (Figure 2B). By day 4, there was an increase in the number of CFUs in the substrate inoculated only with *B. cereus*, and a slight decrease in the number of CFUs in the substrate inoculated with both *B. cereus* and black soldier fly larvae (Figure 2C).

### 3.2. Detection and Quantification of B. cereus DNA within Substrate

**Figure 3:**
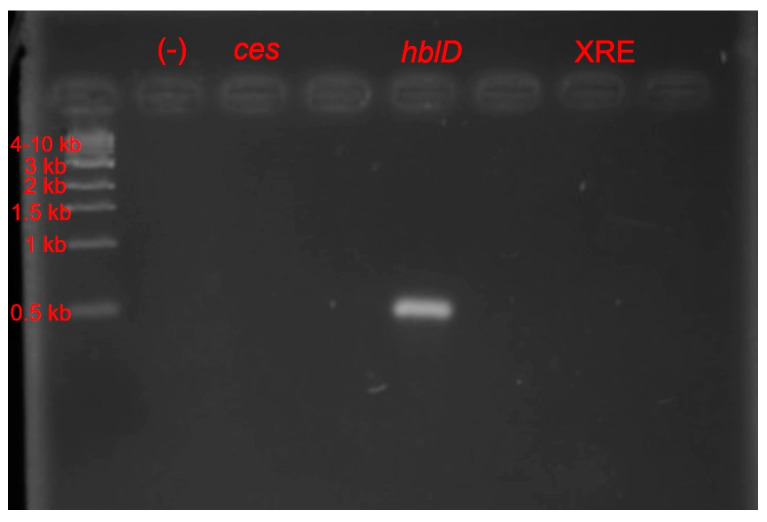
Diagnostic PCR gel indicating positive identification of *hblD* sequence from gDNA extracts of potato substrate containing black soldier fly larvae and *B. cereus*. An electrophoresis gel band at approximately 500 bp indicated presence of the β-hemoysin (*hblD*) sequence unique to *B. cereus* strains. No *ces* and no *XRE* bands were detected in any substrate samples. Lane Ld., 1 kb DNA ladder. Wells in image without labels were not loaded with sample.

Gel electrophoresis of PCR of the β-hemolysin (*hblD*) gene using gDNA from potato substrate confirmed the presence of *B. cereus* in samples inoculated with *B. cereus* (Figure 3; Table 3). At the same time, no cereulide synthase (*ces*) or insecticidal toxin regulator (*XRE*) was detected in these samples. Undiluted gDNA extracted directly from *B. cereus* grown in trypticase soy broth was similarly positive for *hblD*, but negative for *ces* and *XRE*. When diluted, the limit of detection for *hblD* was 1.0 × 10^6^ ng/µL for PCR using either genomic DNA extracted from *B. cereus* or substrate samples.

**Figure 4:**
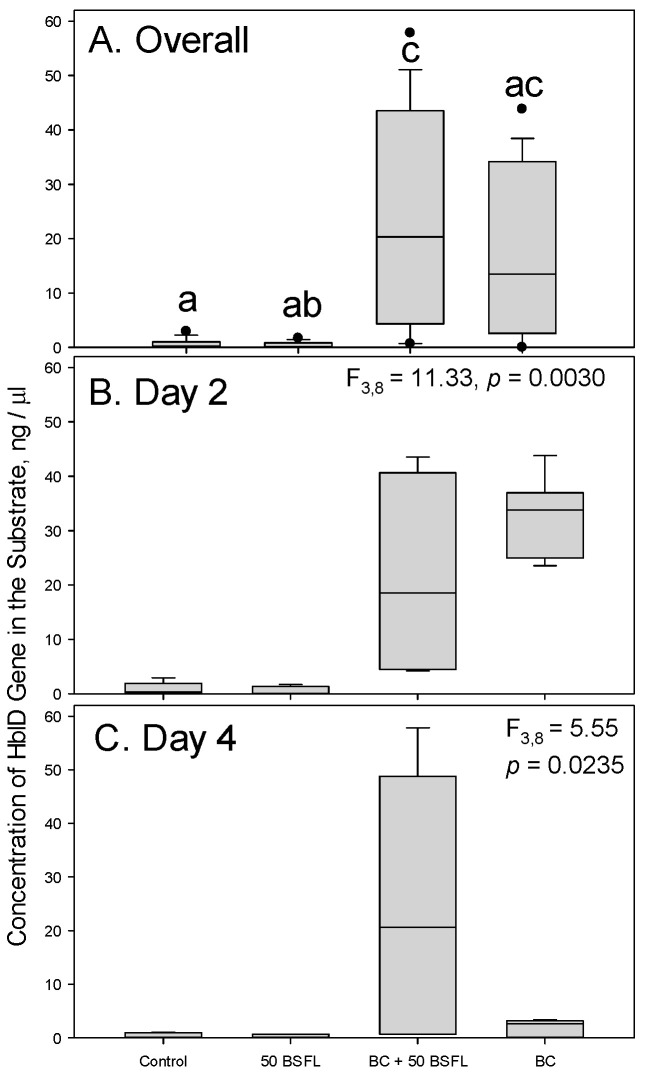
Average total amounts of *hblD* amplified from gDNA extracts of substrate containing *B. cereus* in the presence of 50 black soldier fly larvae using quantitative PCR (qPCR). Control—potato only, BC + 50 BSFL—potato with *B. cereus* and 50 black soldier fly larvae combined, BC—potato with *Bacillus cereus*. (**A**) Overall counts during the entire experiment; (**B**) counts on day 2 of the experiment; (**C**) counts on day 4 of the experiment. Box plots followed by the same lowercase letters indicate treatments that were not significantly different from each other (Tukey tests, *p* < 0.05). Separate F and *p* values for the two sampling days were obtained using the SLICE option (PROC MIXED, SAS Institute, 2021, Cary, NC, USA).

Quantification of the *hblD* sequences within the substrates indicated that both treatment (df = 3,8, F = 9.39, *p* = 0.0053) and day (df = 1,8, F = 9.90, *p* = 0.0137) had significant effects on the concentration of *hblD*. The interaction effects between treatment and day (df = 3,8, F = 4.96, *p* = 0.0313) also had a significant effect on DNA concentration over time. The substrate that was inoculated with both *B. cereus* and black soldier fly larvae consistently had more *hblD* than substrates subjected to the other treatments (Figure 4). For the substrate with *B. cereus* alone, there was a dramatic drop in *hblD* concentration between days 2 and 4 of the experiment (Figure 4B,C). The substrates that were not inoculated with *B. cereus* had nearly undetectable levels of *hblD* (Figure 4B,C).

## 4. Discussion

Suppression of pathogenic microorganisms is commonly reported for a variety of substrates inhabited by black soldier fly larvae and represents an additional benefit of using them for recycling organic wastes [8,9,10,24]. In the present study, however, we did not detect any reduction in *B. cereus* abundance in potato substrate in the presence of the black soldier fly larvae. To the contrary, the culturable amount of *B. cereus* increased when the substrate was inoculated with 50 black soldier fly larvae (Figure 2 and Figure 3B), and so did the abundance of the *hblD* marker gene (Figure 4). Such an enhancement of bacterial growth has been seldom reported, although Erickson et al. [25] (p. 688) observed improved survival of *Escherichia coli* O157:H7 in hog manure in the presence of black soldier fly larvae. There is also speculation that the presence of certain *Bacillus* sp. may be ecologically favored during black soldier fly larvae development [26].

Adding 100 black soldier fly larvae had a less pronounced overall effect on *B. cereus* or total bacteria, as the overall number of CFUs in that substrate was comparable to the substrate inoculated with *B. cereus* but lacking larvae (Figure 1 and Figure 2). Since the initial exacerbation of *B. cereus* was detected at two days but mitigated at five days in this treatment, there may be potential for suppression of *B. cereus* at higher larval densities or on longer time spans. The larval densities utilized in these experiments were lower than previously published ratios for soldier fly larvae relative to growth substrates [27,28,29], which allowed for clarity to establish the observed phenomenon. This may indicate a potential risk when using suboptimal larval density for undesirable increases in contaminating pathogenic bacteria. More research should explore these possibilities with *B. cereus* and other spore-forming bacteria.

When *B. cereus* was applied at the concentration of approximately 1.0 × 10^8^ CFU/mL in the first experiment, the exacerbating effect was observed in the samples collected after five days of incubation (Figure 2C), but not after two days of incubation (Figure 2B). The opposite was true when *B. cereus* was applied at the lower concentration of approximately 4.0 × 10^7^ CFU/mL in the second experiment (Figure 3B,C). Both black soldier flies [30] (p. 29) and *B. cereus* [17] (p. 494) are known to produce amylase, which reduces the length of polysaccharide chains in potato tubers and permits their digestion. This may at least partially explain facilitation of bacterial growth by the black soldier fly larvae introduced at a lower density. The larvae have extraintestinal digestion; therefore, their releasing digestive enzymes, including amylase, into potato substrate may benefit *B. cereus* [31,32]. This may be more important for the bacteria inoculated at a lower concentration and, consequently, producing less enzymes of their own. Therefore, the effect was manifested earlier in the second experiment.

At a higher larval density, positive effects of possible synergism may have been counterbalanced by the negative effects of competition. Both larvae and the corresponding microbial community are in constant competition for resources originating from the substrate matter [33,34]. Additionally, concentration of compounds, such as antimicrobial peptides or lysozymes, which both play a key role in larval immunity, is likely to increase when more black soldier fly larvae are present [5,35]. Furthermore, the ability of larvae to suppress different infectious agents can be altered if changes in microbiome communities occur [36,37]. Previous studies involving black soldier fly development in the presence of pathogenic bacteria suggest that communal residents of the microbiome play a significant role in pathogenic suppression in addition to the antimicrobial factors produced by the larvae themselves [33,34]. In the current study, doubling the population of BSFL from 50 to 100 larvae likely led to increased populations of antagonistic microorganisms, putting *B. cereus* at additional disadvantage and mitigating the initial increase in pathogen load. Future studies should also assess the possible negative effects of *B. cereus* on BSF physiology through assessment of weight gain and survival metrics to provide more important context for these observations.

Changes in the levels of *hblD* sequence amplicons suggested that the presence of black soldier fly larvae may produce a conditioning effect for *B. cereus* that could lead to potential growth of endospores present in non-sterile substrates. This conditioning may be the result of amylase production during both larval and bacterial metabolism [17,30] (p. 494, p. 29), which reduces the length of polysaccharide chains within potato tubers and permits digestion by larvae. Black soldier fly larvae used in our experiments did not appear to harbor *B. cereus* themselves because it was absent from the substrate that was inoculated only with larvae (Figure 3 and Figure 4).

The significant decrease in *hblD* amplification for day 4 of the experiment in the absence of black soldier fly larvae is expectedly incongruent with the data collected from enumeration of *B. cereus* colonies. As *B. cereus* is a known endospore-forming bacteria, it is likely that the number of vegetative cells were reduced (and replaced with endospores) as time elapsed during experimentation. Timing of sporulation has been reported to be quite variable but can have a time as short as one day in defined growth media [38,39]. Endospores return to active growth when inoculated into a new nutrient-rich medium (such as BCA), and as such colonies may be derived from either endospores or vegetative cells. However, extraction of DNA from endospores requires specific methodology [40] (p. 18), and no singular method for DNA extraction is efficient for both endospores and vegetative cells of Gram-positive and Gram-negative bacteria [41]. This leads to biases in amplification from those templates and likely explains a reduction in observed *HblD* in substrates only amended with *B. cereus* when they were deprived of the possible synergism with BSFL. Additional experiments that explore the composition of *B. cereus* endospores relative to vegetative cells at later time points during feeding are an important next step in understanding the ecology of spore-forming bacteria and black soldier fly larvae.

Our results highlight that interactions between black soldier fly larvae and microbial communities are complicated, dynamic, and likely to be specific to particular substrates and conditions [36,37]. While well-documented suppression of several common pathogens is a definite benefit of using the larvae in waste management, they should not be treated as biological sterilizers that automatically make any substrate safe from contamination by pathogenic microorganisms [42,43]. Safety issues should always be an important consideration when employing this valuable technology.

## Figures and Tables

**Table 1:** Information for primer sets used in diagnostic and quantitative PCR.

Target	Primer Sequence (5′-3′)	Function	Amplicon Length	Reference
*hblD*	GTTAGATACAGCGAAGCCAC	Forward Primer	465 bp	[19]
	CCGCCAGTTACAACAATA	Reverse Primer		
*ces*	CGCCGAAAGTGATTATACCAA	Forward Primer	103 bp	[20]
	TATGCCCCGTTCTCAAACTG	Reverse Primer		
*XRE*	AAGATATTGCAAGCGGTAAGAT	Forward Primer	270 bp	[21]
	GTTTTGTTTCAGCATTCCAGTAA	Reverse Primer		

**Table 2:** PCR reaction conditions for each primer set.

Primer Set	Melting Temp	Annealing Temp	Elongation Temp	Primer Concentration	Amplicon Length	Cycle Number
*hblD*	95 °C Initial = 10 min Cycle = 30 s	52.2 °C Cycle = 30 s	72 °C Cycle = 30 s Final = 10 min	0.2 µM	465 bp	35
*Ces*	95 °C Initial = 2 min Cycle = 30 s	60 °C Cycle = 60 s	72 °C Cycle = 30 s Final = 5 min	0.3 µM	103 bp	35
*XRE*	95 °C Initial = 5 min Cycle = 30 s	49 °C Cycle = 30s	72 °C Cycle = 30 s Final = 5min	0.3 µM	246 bp	35

**Table 3:** Summary of the gel electrophoresis results indicating the presence of *hblD* within each treatment. The expected limit of detection was 1.0 × 10^6^ ng/µL.

Treatment	Replicate	Day 2	Day 4
Substrate	A	−	−
	B	−	−
	C	−	−
Larvae	A	−	−
	B	−	−
	C	−	−
Larvae and Pathogen	A	+	+
	B	+	+
	C	+	+
Pathogen	A	+	+
	B	−	−
	C	−	−

## Data Availability

Publicly available datasets were analyzed in this study. This data can be found here: http://andreialyokhin.com/research_data/Bacillus_cereus_data.xlsx, accessed on 18 March 2023.

## References

[1] Purschke B., Scheibelberger R., Axmann S., Adler A., Jäger H. (2017). Impact of Substrate Contamination with Mycotoxins, Heavy Metals and Pesticides on the Growth Performance and Composition of Black Soldier Fly Larvae (*Hermetia illucens*) for Use in the Feed and Food Value Chain. Food Addit. Contam. Part A.

[2] Vandeweyer D., De Smet J., Van Looveren N., Van Campenhout L. (2021). Biological Contaminants in Insects as Food and Feed. J. Insects Food Feed.

[3] Bonelli M., Bruno D., Brilli M., Gianfranceschi N., Tian L., Tettamanti G., Caccia S., Casartelli M. (2020). Black Soldier Fly Larvae Adapt to Different Food Substrates through Morphological and Functional Responses of the Midgut. Int. J. Mol. Sci..

[4] Bruno D., Montali A., Mastore M., Brivio M.F., Mohamed A., Tian L., Grimaldi A., Casartelli M., Tettamanti G. (2021). Insights Into the Immune Response of the Black Soldier Fly Larvae to Bacteria. Front. Immunol..

[5] Liu Q., Tomberlin J.K., Brady J.A., Sanford M.R., Yu Z. (2008). Black Soldier Fly (Diptera: Stratiomyidae) Larvae Reduce *Escherichia coli* in Dairy Manure. Environ. Entomol..

[6] Bessa L.W., Pieterse E., Marais J., Dhanani K., Hoffman L.C. (2021). Food Safety of Consuming Black Soldier Fly (*Hermetia illucens*) Larvae: Microbial, Heavy Metal and Cross-Reactive Allergen Risks. Foods.

[7] Choi W.-H., Yun J.-H., Chu J.-P., Chu K.-B. (2012). Antibacterial Effect of Extracts of *Hermetia illucens* (Diptera: Stratiomyidae) Larvae against Gram-Negative Bacteria: *Hermetia illucens* Antibacterial Activity. Entomol. Res..

[8] Park S.-I., Yoe S.M. (2017). Defensin-like Peptide3 from Black Solder Fly: Identification, Characterization, and Key Amino Acids for Anti-Gram-Negative Bacteria: Defensin-like Peptide3 from *H. illucens*. Entomol. Res..

[9] Kinney M., Moyet M., Bernard E., Alyokhin A. (2022). Suppression of Methicillin-Resistant Staphylococcus Aureus and Reduction of Other Bacteria by Black Soldier Fly Larvae Reared on Potato Substrate. Microbiol. Spectr..

[10] Kim M.-J., Han J., Park J.-S., Lee J.-S., Lee S.-H., Cho J.-I., Kim K.-S. (2015). Various Enterotoxin and Other Virulence Factor Genes Widespread among *Bacillus cereus* and *Bacillus thuringiensis* Strains. J. Microbiol. Biotechnol..

[11] Oh M.-H., Ham J.-S., Seol K.-H., Jang A.-R., Lee S.-G., Lee J.-M., Park B.-Y., Kang E.-S., Kwon K.-S., Hwang I.-G. (2011). Growth Profile and Toxigenicity of *Bacillus cereus* in Ready-to-Eat Food Products of Animal Origin. Korean J. Food Sci. Anim. Resour..

[12] Huang Y., Flint S.H., Yu S., Ding Y., Palmer J.S. (2021). Phenotypic Properties and Genotyping Analysis of *Bacillus cereus* Group Isolates from Dairy and Potato Products. LWT.

[13] Heini N., Stephan R., Ehling-Schulz M., Johler S. (2018). Characterization of *Bacillus cereus* Group Isolates from Powdered Food Products. Int. J. Food Microbiol..

[14] Tallent S.M., Hait J.M., Ferguson M. (2018). Comparative Study of Tempo BC Automated MPN for the Enumeration of *Bacillus cereus* Group in Food. J. Food Saf..

[15] Fangio M.F., Roura S.I., Fritz R. (2010). Isolation and Identification of *Bacillus* Spp. and Related Genera from Different Starchy Foods. J. Food Sci..

[16] Vaikundamoorthy R., Rajendran R., Selvaraju A., Moorthy K., Perumal S. (2018). Development of Thermostable Amylase Enzyme from *Bacillus cereus* for Potential Antibiofilm Activity. Bioorganic Chem..

[17] Sheppard D.C., Tomberlin J.K., Joyce J.A., Kiser B.C., Sumner S.M. (2002). Rearing Methods for the Black Soldier Fly (Diptera: Stratiomyidae). J. Med. Entomol..

[18] Procop G.W., Church D.L., Hall G.S., Janda W.M., Koneman E.W., Schreckenberger P.C., Woods G.L. (2017). Koneman’s Color Atlas and Textbook of Diagnostic Microbiology.

[19] Zhang Z., Feng L., Xu H., Liu C., Shah N.P., Wei H. (2016). Detection of Viable Enterotoxin-Producing *Bacillus cereus* and Analysis of Toxigenicity from Ready-to-Eat Foods and Infant Formula Milk Powder by Multiplex PCR. J. Dairy Sci..

[20] Fricker M., Messelhäußer U., Busch U., Scherer S., Ehling-Schulz M. (2007). Diagnostic Real-Time PCR Assays for the Detection of Emetic *Bacillus cereus* Strains in Foods and Recent Food-Borne Outbreaks. Appl Environ. Microbiol.

[21] Wei S., Chelliah R., Park B.-J., Kim S.-H., Forghani F., Cho M.S., Park D.-S., Jin Y.-G., Oh D.-H. (2019). Differentiation of *Bacillus thuringiensis* from *Bacillus cereus* Group Using a Unique Marker Based on Real-Time PCR. Front. Microbiol..

[22] Conover W.J., Iman R.L. (1981). Rank Transformations as a Bridge between Parametric and Nonparametric Statistics. Am. Stat..

[23] Wobbrock J.O., Findlater L., Gergle D., Higgins J.J. (2011). The Aligned Rank Transform for Nonparametric Factorial Analyses Using Only ANOVA Procedures. Proceedings of the SIGCHI Conference on Human Factors in Computing Systems.

[24] Bernard E., Villazana J., Alyokhin A., Rose J. (2020). Colonisation of Finfish Substrate Inhabited by Black Soldier Fly Larvae by Blow Flies, Bacteria, and Fungi. J. Insects Food Feed.

[25] Erickson M.C., Islam M., Sheppard C., Liao J., Doyle M.P. (2004). Reduction of *Escherichia coli* O157:H7 and *Salmonella enterica* Serovar Enteritidis in Chicken Manure by Larvae of the Black Soldier Fly. J. Food Prot..

[26] Callegari M., Jucker C., Fusi M., Leonardi M.G., Daffonchio D., Borin S., Savoldelli S., Crotti E. (2020). Hydrolytic Profile of the Culturable Gut Bacterial Community Associated with *Hermetia illucens*. Front. Microbiol..

[27] Diener S., Zurbrügg C., Tockner K. (2009). Conversion of Organic Material by Black Soldier Fly Larvae: Establishing Optimal Feeding Rates. Waste Manag. Res..

[28] Parra Paz A.S., Carrejo N.S., Gómez Rodríguez C.H. (2015). Effects of Larval Density and Feeding Rates on the Bioconversion of Vegetable Waste Using Black Soldier Fly Larvae *Hermetia illucens* (L.), (Diptera: Stratiomyidae). Waste Biomass Valoriz..

[29] Broeckx L., Frooninckx L., Slegers L., Berrens S., Noyens I., Goossens S., Verheyen G., Wuyts A., Van Miert S. (2021). Growth of Black Soldier Fly Larvae Reared on Organic Side-Streams. Sustainability.

[30] Rabani V., Cheatsazan H., Davani S. (2019). Proteomics and Lipidomics of Black Soldier Fly (Diptera: Stratiomyidae) and Blow Fly (Diptera: Calliphoridae) Larvae. J. Insect Sci..

[31] Goodbrod J.R., Goff M.L. (1990). Effects of Larval Population Density on Rates of Development and Interactions between Two Species of *Chrysomya* (Diptera: Calliphoridae) in Laboratory Culture. J. Med. Entomol..

[32] Green P.W.C., Simmonds M.S.J., Blaney W.M. (2003). Diet Nutriment and Rearing Density Affect the Growth of Black Blowfly Larvae, *Phormia regina* (Diptera: Calliphoridae). Eur. J. Entomol..

[33] Tegtmeier D., Hurka S., Mihajlovic S., Bodenschatz M., Schlimbach S., Vilcinskas A. (2021). Culture-Independent and Culture-Dependent Characterization of the Black Soldier Fly Gut Microbiome Reveals a Large Proportion of Culturable Bacteria with Potential for Industrial Applications. Microorganisms.

[34] Wu N., Liang J., Wang X., Xie S., Xu X. (2021). Copper Stimulates the Incidence of Antibiotic Resistance, Metal Resistance and Potential Pathogens in the Gut of Black Soldier Fly Larvae. J. Environ. Sci..

[35] Candian V., Meneguz M., Tedeschi R. (2023). Immune Responses of the Black Soldier Fly *Hermetia illucens* (L.) (Diptera: Stratiomyidae) Reared on Catering Waste. Life.

[36] Wynants E., Frooninckx L., Crauwels S., Verreth C., De Smet J., Sandrock C., Wohlfahrt J., Van Schelt J., Depraetere S., Lievens B. (2019). Assessing the Microbiota of Black Soldier Fly Larvae (*Hermetia illucens*) Reared on Organic Waste Streams on Four Different Locations at Laboratory and Large Scale. Microb. Ecol..

[37] Schreven S.J.J., de Vries H., Hermes G.D.A., Zeni G., Smidt H., Dicke M., van Loon J.J.A. (2022). Black Soldier Fly Larvae Influence Internal and Substrate Bacterial Community Composition Depending on Substrate Type and Larval Density. Appl. Environ. Microbiol..

[38] de Vries Y.P., Hornstra L.M., de Vos W.M., Abee T. (2004). Growth and Sporulation of *Bacillus cereus* ATCC 14579 under Defined Conditions: Temporal Expression of Genes for Key Sigma Factors. Appl. Environ. Microbiol..

[39] Bressuire-Isoard C., Broussolle V., Carlin F. (2018). Sporulation Environment Influences Spore Properties in Bacillus: Evidence and Insights on Underlying Molecular and Physiological Mechanisms. FEMS Microbiol. Rev..

[40] Knüpfer M., Braun P., Baumann K., Rehn A., Antwerpen M., Grass G., Wölfel R. (2020). Evaluation of a Highly Efficient DNA Extraction Method for Bacillus Anthracis Endospores. Microorganisms.

[41] Roopnarain A., Mukhuba M., Adeleke R., Moeletsi M. (2017). Biases during DNA Extraction Affect Bacterial and Archaeal Community Profile of Anaerobic Digestion Samples. 3 Biotech.

[42] Awasthi M.K., Liu T., Awasthi S.K., Duan Y., Pandey A., Zhang Z. (2020). Manure Pretreatments with Black Soldier Fly *Hermetia illucens* L. (Diptera: Stratiomyidae): A Study to Reduce Pathogen Content. Sci. Total Environ..

[43] Liu T., Klammsteiner T., Dregulo A.M., Kumar V., Zhou Y., Zhang Z., Awasthi M.K. (2022). Black Soldier Fly Larvae for Organic Manure Recycling and Its Potential for a Circular Bioeconomy: A Review. Sci. Total Environ..

